# The life cycle of platelet granules

**DOI:** 10.12688/f1000research.13283.1

**Published:** 2018-02-28

**Authors:** Anish Sharda, Robert Flaumenhaft

**Affiliations:** 1 Division of Hemostasis and Thrombosis, Department of Medicine, Beth Israel Deaconess Medical Center, Harvard Medical School, Boston, USA

**Keywords:** Platelets, Granule, Exocytosis, Platelet activation

## Abstract

Platelet granules are unique among secretory vesicles in both their content and their life cycle. Platelets contain three major granule types—dense granules, α-granules, and lysosomes—although other granule types have been reported. Dense granules and α-granules are the most well-studied and the most physiologically important. Platelet granules are formed in large, multilobulated cells, termed megakaryocytes, prior to transport into platelets. The biogenesis of dense granules and α-granules involves common but also distinct pathways. Both are formed from the *trans*-Golgi network and early endosomes and mature in multivesicular bodies, but the formation of dense granules requires trafficking machinery different from that of α-granules. Following formation in the megakaryocyte body, both granule types are transported through and mature in long proplatelet extensions prior to the release of nascent platelets into the bloodstream. Granules remain stored in circulating platelets until platelet activation triggers the exocytosis of their contents. Soluble *N*-ethylmaleimide-sensitive factor attachment protein receptor (SNARE) proteins, located on both the granules and target membranes, provide the mechanical energy that enables membrane fusion during both granulogenesis and exocytosis. The function of these core fusion engines is controlled by SNARE regulators, which direct the site, timing, and extent to which these SNAREs interact and consequently the resulting membrane fusion. In this review, we assess new developments in the study of platelet granules, from their generation to their exocytosis.

## Introduction

Platelets are anucleate, discoid-shaped blood cells essential for hemostasis, which serves to maintain the integrity of the vasculature upon injury. The functional role of platelets has expanded in recent years to include processes such as inflammation, innate immunity, growth and development, angiogenesis, wound healing, and cancer metastasis ^1^. Platelet granule exocytosis is central to platelet function and participates in the full repertoire of platelet activities. Platelets contain at least three major types of granules— **α**-granules, dense granules, and lysosomes—which carry distinct cargos and vary in biogenesis, trafficking, and exocytosis. In addition, platelets have peroxisomes and recently described T granules. This review focuses on the biogenesis of platelet **α**- and dense granules and mechanisms of their exocytosis.

## Platelet granules

α-Granules are unique to platelets and are the most abundant of the platelet granules, numbering 50–80 per platelet ^2^. These granules measure 200–500 nm in diameter and account for about 10% of platelet volume. They contain mainly proteins, both membrane-associated receptors (for example, **α**IIbβ3 and P-selectin) and soluble cargo (for example, platelet factor 4 [PF4] and fibrinogen). Proteomic studies have identified more than 300 soluble proteins that are involved in a wide variety of functions, including hemostasis (for example, von Willebrand factor [VWF] and factor V), inflammation (for example, chemokines such as CXCL1 and interleukin-8), and wound healing (for example, vascular endothelial growth factor [VEGF] and fibroblast growth factor [FGF]) ^3^. The classic representation of **α**-granules as spherical organelles with a peripheral limiting membrane, a dense nucleoid, and progressively lucent peripheral zones on transmission electron microscopy is probably simplistic and may be in part a preparation artifact. Electron tomography with three-dimensional reconstruction of platelets is notable for a significant percentage of tubular **α**-granules that generally lack VWF ^4^. More recent work using transmission electron microscopy and freeze substitution dehydration of resting platelets shows that **α**-granules are ovoid with a generally homogeneous matrix and that tubes form from α-granules upon activation ^5^. Thus, whether or not there exists significant structural heterogeneity among α-granules remains to be completely resolved. **α**-Granule exocytosis is evaluated primarily by plasma membrane expression of P-selectin (CD62P) by flow cytometry or estimation of the release of PF4, VWF, or other granule cargos ^6^.

Dense granules (also known as δ-granules) are the second most abundant platelet granules, with 3–8 per platelet. They measure about 150 nm in diameter ^2^. These granules, unique to the platelets, are a subtype of lysosome-related organelles (LROs), a group that also includes melanosomes, lamellar bodies of the type II alveolar cells, and lytic granules of cytotoxic T cells ^7^. Dense granules mainly contain bioactive amines (for example, serotonin and histamine), adenine nucleotides, polyphosphates, and pyrophosphates as well as high concentrations of cations, particularly calcium. These granules derive their name from their electron-dense appearance on whole mount electron microscopy, which results from their high cation concentrations ^8^. Dense granule exocytosis is typically evaluated by ADP/ATP release by using luciferase-based luminescence techniques, release of preloaded [ ^3^H] serotonin, or membrane expression of lysosome-associated membrane protein 2 (LAMP2) or CD63 by flow cytometry ^6^.

Other platelet granules have been described. Platelets contain about 1–3 lysosomes per platelet and peroxisomes, the platelet-specific function of which remains unclear. Lysosomal exocytosis is typically evaluated by estimation of released lysosomal enzymes such as beta hexosaminidase. An electron-dense granule defined by the presence of Toll-like receptor 9 (TLR9) and protein disulfide isomerase (PDI), termed the T granule, has also been described, although its existence remains controversial ^9^. PDI and other platelet-borne thiol isomerases have been reported to be packaged within a non-granular compartment derived from the megakaryocyte endoplasmic reticulum (ER), which may be associated with the dense tubular system ^10, 11^.

## Biogenesis of platelet granules

Formation of platelet granules begins in megakaryocytes, but maturation continues in circulating platelets ^12, 13^. Human platelet granule deficiency syndromes, also referred to as storage pool disorders, and their related murine models have been a major source of study of platelet granulogenesis. Gray platelet syndrome (GPS), an **α**-granule deficiency disorder, and Hermansky–Pudlak syndrome (HPS), a group of dense granule deficiency syndromes, are two such examples. GPS platelets contain normal dense granules, whereas HPS6 platelets contain normal **α**-granules, which suggests that these granules have distinct pathways of biogenesis ^7, 14, 15^. In recent years, many inherited disorders due to defects in transcription factors such as *RUNX1*, *GATA1*, *FLl1*, *GFI1b*, and *ETV6* have been found to impact megakaryopoiesis and impair platelet production and maturation ^16– 21^. Many of these disorders are associated with one or more granule deficiency states and have helped elucidate the role of these genes in platelet granulogenesis.

### α-Granule biogenesis

**Figure 1.**
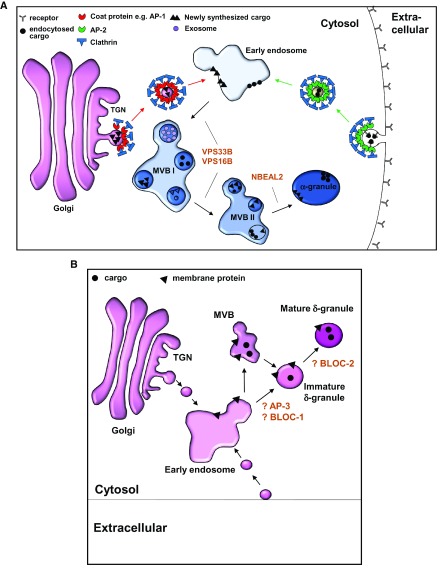
Working models of platelet α-granule and dense granule formation in megakaryocytes. ( **A**) **α**-Granules derive from two major pathways: synthetic and endocytic. The synthetic pathway originates at the *trans*-Golgi network (TGN). Soluble clathrin molecules recruited to the TGN self-assemble into a lattice structure and interact with coat proteins, presumed to be adaptor protein 1 (AP1), to form clathrin-coated pits. These pits invaginate to bud off early membrane-bound vesicles that are ultimately directed to early endosomes. Endocytic vesicles originate similarly at the plasma membrane employing adaptor protein 2 (AP2) and ultimately merge into early endosomes. **α**-Granules mature in multivesicular bodies (MVBs), a process that requires proteins VPS33B, VPS16B, and NBEAL2. ( **B**) Dense (δ) granules are lysosomal-related organelles, which are derived from the endosomal compartment. The current understanding of biogenesis of dense granule is highly speculative and was extrapolated from the biogenesis of melanosomes. Early endosomes provide input for developing dense granules, which may mature in MVBs. In melanosomes, BLOC1 is required for the exit of tubular structures carrying cargo from the endosomes, which are directed to the developing melanosomes by BLOC2. Alternatively, cargoes can be directed to developing dense granules by an AP3-dependent pathway, which may or may not require BLOC2. BLOC, biogenesis of lysosome-related organelles complex.

**α**-Granule proteins derive from both synthetic and endocytic pathways ^22^. Synthetic pathways traffic translated proteins from ER to **α**-granules. The endocytic pathway enables megakaryocytes and mature platelets to acquire plasma proteins by the process of endocytosis at the plasma membrane ^23^. Multiple individual proteins and protein complexes mediate trafficking of these separate pathways ( Figure 1A). Such proteins include coat proteins such as clathrin, adaptor proteins AP1 and AP2, and proteins required for vesicle trafficking, including soluble *N*-ethylmaleimide-sensitive factor (NSF) attachment protein receptor (SNARE) proteins, SNARE regulators, particularly Sec1/Munc18 proteins, and small GTPases such as Rabs. As a first step, soluble clathrin molecules recruited to either *trans*-Golgi network (TGN) or plasma membrane self-assemble into a lattice structure and interact with APs to form clathrin-coated pits. Platelets contain clathrin-associated adaptor proteins AP1, AP2, and AP3 ^24^. Since AP2 localizes only to plasma membrane where it functions in the endocytotic pathway and AP3 is critical for lysosomal and LRO trafficking, the deficiency of which leads only to dense granule deficiency as in HPS subtype 2, AP1 is assumed to be employed by the synthetic pathway in **α**-granule biogenesis, although there is no direct evidence for this or for the role of other coat proteins such as COPI in **α**-granule biogenesis ^25^. Vesicles carrying **α**-granule cargo budding off from either TGN or plasma membrane are subsequently directed to multivesicular bodies (MVBs) via endosomes ^26^.

MVBs are transient late endosomal structures that contain internal vesicles formed from inward budding of the limiting membrane of the endosome ^27^. Initially assumed to only direct proteins to be degraded in the lysosomes, these structures are now known to have multiple other functions, including granule trafficking in various cell types. MVBs serve as an intermediate stage of granulogenesis in megakaryocytes ^26^. **α**-Granule cargoes from both synthetic and endocytic pathways can be identified in MVBs ^26^. Both **α**- and dense granules mature from MVBs but use distinct machinery ^26^. For example, defects in VPS33B and NBEAL2 lead only to **α**-granule deficiency but not to dense granule defects. VPS33B, a Sec1/Munc18 protein deficient in arthrogryposis, renal dysfunction, and cholestasis (ARC) syndrome, was the first protein involved in **α**-granule biogenesis to be identified ^28, 29^. VPS16B, its partner, works in association ^30^. Although many platelet-specific details remain poorly understood, the SNARE binding function of VPS33 and VPS16 in vesicular trafficking as a component of two large protein complexes—class C core vacuole/endosome/tethering (CORVET), containing isoforms VPS33B and VPS16B, and homotypic fusion and protein sorting (HOPS), containing isoforms VPS33A and VPS16A—has been characterized in yeast ^31^. The other proteins of these complexes have membrane-, AP-, and Rab-binding properties, thus bringing together the basic machinery required for endosomal maturation. NBEAL2 deficiency as a cause of GPS was first described in 2011 ^14, 32, 33^. *Nbeal2* ^-/-^ mice exhibit a phenotype similar to that of patients with GPS, including macrothrombocytopenia, splenomegaly, and myelofibrosis, but the exact molecular function of NBEAL2 is not known ^34^. It acts at a later state of **α**-granule development, independently of VPS33B, as *Nbeal2* ^-/-^ platelets express some P-selectin that externalizes upon platelet activation. NBEAL2 is under direct transcriptional control of *GATA1*, a mutation in which results in a syndrome similar to GPS, in addition to myelodysplasia ^35^. It is one of the nine BEACH (beige and Chediak-Higashi) domain-containing proteins, hypothesized to be scaffolds for fission and fusion membrane events ^36^. Genetic defects in Chediak-Higashi syndrome 1 ( *CHS1*), another member of this family, lead to platelet dysfunction secondary to dense granule deficiency in addition to immunodeficiency and other manifestations ^36^.

Protein sorting and packaging into the developing **α**-granule occur via varying mechanisms dependent on the protein type. Many membrane proteins, such as P-selectin, contain signal peptides that direct them to the developing granule ^37^. Notably, P-selectin uses distinct signal peptides for trafficking to the **α**-granules in platelets and Weibel–Palade bodies in endothelial cells ^38^. Another mechanism is protein aggregation, which is employed by large soluble proteins such as multimerin and VWF ^39, 40^. VWF self-assembles into large homoaggregates that ultimately form tubular structures occupying a distinct sub-compartment within **α**-granules ^41^. Sorting sequences contribute to trafficking of many smaller soluble proteins to **α**-granules. PF4 is one such protein that has a four-amino acid sequence within its hydrophilic loop that directs it to the maturing **α**-granule ^42^. Other examples of small proteins that employ sorting sequences are RANTES and NAP2. Cationic glycosaminoglycans within **α**-granules may also serve to retain these small chemokines ^43^. Exogenous proteins are trafficked through an endocytic pathway into **α**-granules via either receptor-mediated endocytosis or pinocytosis. Fibrinogen, which is internalized via integrin **α**IIbβ _3_, is a classic example of this route, which subsequently uses adaptor protein Disabled-2 for formation of clathrin-coated vesicles ^44, 45^. Proteins that are incorporated into platelets via pinocytosis include immunoglobulins as well as angiogenesis regulators such as VEGF, endostatin, and FGF ^23, 46^. Vesicle-associated membrane protein 3 (VAMP-3), a v-SNARE (discussed below), regulates platelet endocytosis. VAMP-3 ^-/-^ platelets show impaired **α**IIbβ _3_-mediated fibrinogen uptake ^47^. In addition, loss of VAMP-3 impairs trafficking of both endocytosed and pinocytosed cargo between Rab4 (early endosomes) and Rab11 (recycling endosomes) positive compartments, although its mechanism remains unclear. Endocytosis of plasma proteins starts in megakaryocytes but continues in mature circulating platelets. For example, platelets from patients with complete factor V deficiency endocytose and release factor V supplemented in transfused plasma for prolonged periods greater than the half-life of factor V ^48^.

### Dense granule biogenesis

Dense granules are platelet-specific LROs ^7^. These granules are distinct from classic secretory granules in that they are derived from the endosomal system instead of directly from TGN ( Figure 1B). They also share some characteristics with lysosomes as their intra-granular pH is acidic and they possess lysosome-resident proteins, such as the tetraspanin CD63. However, CD63 is not restricted to dense granules in platelets, and the lack of other specific cargoes that can be followed biosynthetically has made evaluation of dense granule biogenesis challenging. There is evidence that early endosomes contribute to dense granule biogenesis ^49^. In addition, like **α**-granules, dense granules are believed to be sorted in MVBs, although the only direct evidence of this is the accumulation of CD63 and serotonin in MVBs in megakaryocytes ^50^. HPS and related disorders together with their murine counterparts have served as a great source of understanding of biogenesis of LROs, and melanosomes are the prototype organelle that has been studied.

In total, at least 10 different HPS genes encode subunits of four distinct ubiquitously present protein complexes: adaptor protein-3 (AP3) and biogenesis of lysosome-related organelles complex (BLOC) 1, 2, and 3 ^51– 54^. These complexes localize mainly to the endosomal compartment and are essential for biogenesis of LROs. Deficiency or alteration in these proteins results in two common manifestations: albinism due to abnormal melanogenesis and a bleeding disorder due to dense granule deficiency. Some HPS subtypes display other manifestations, such as pulmonary fibrosis, inflammatory bowel disease, and immunodeficiency ^51^. Functions of these individual proteins and protein complexes are being understood with increasing detail. In melanosomes, BLOC1 (complex of HPS7, HPS8, HPS9, Muted, Cappuccino, Snapin, BLOS2, and BLOS3) is required for the exit of melanosome cargoes from endosomes into tubular transport carriers ^55^. BLOC2 (complex of HPS3, HPS5, and HPS6) directs these carriers specifically to the melanosomes. Alternatively, cargoes can be directed into developing melanosomes in an AP3-dependent pathway, which in turn can be BLOC1-independent or -dependent ^55– 58^. BLOC3 (complex of HPS1 and 4) functions after cargo delivery in pathways out of melanosomes, specifically in retrieval and recycling of the BLOC1-dependent v-SNARE VAMP-7 ^59^. Owing to concurrence of albinism and dense granule deficiency in HPS, pathways similar to those described above are thought to function in dense granule biogenesis in megakaryocytes, although there is no direct evidence. The exact molecular functions of many of the HPS and related proteins are also being characterized, mainly in melanosomes. HPS9, or Pallidin, a component of BLOC1, is known to interact with syntaxin 13, a SNARE protein involved in vesicle membrane fusion during trafficking ^60^. BLOC2 constituents HPS3 and HPS6 have been described to bind clathrin and dynactin p150Glued, respectively ^61, 62^. BLOC3 functions as a guanine nucleotide exchange factor for cell type-specific Rab GTPases, such as Rab32 and Rab38 in melanocytes ^63, 64^. A direct role of Rab32 and Rab38 in dense granule biogenesis in megakaryocytes has also been implicated ^13, 64^. *RUNX1* mutations lead to dense granule but not **α**-granule deficiency due to dysregulation of Pallidin (HPS9) transcription ^65^.

Dense granule contents, such as bioactive amines and adenine nucleotides, are transported into the maturing dense granules via specific membrane pumps, such as vesicular nucleotide transporter (VNUT), which has been proposed as a candidate for ADP and ATP accumulation in dense granules, and multidrug resistance-associated protein 4 (MRP4), which uptakes cAMP into dense granules ^66– 68^. MRP4 ^-/-^ mice show significant platelet dysfunction due to cytosolic accumulation of cAMP and lack of cAMP in dense granules, as do inhibitors of MRP4, such as probenecid ^67, 69^. York platelet syndrome is characterized by thrombocytopenia and striking giant electron-opaque organelles. It is caused by a calcium-selective release-activated calcium (CRAC) channelopathy, which results in defective calcium storage ^70^.

## Platelet granule exocytosis

Platelet granule exocytosis is a classic example of regulated secretion. Upon agonist stimulation, cargo stored in platelet granules is released, and rates and extent are dependent on the stimulation strength ^71^. Dense granule exocytosis is fastest and most sensitive to agonists, whereas lysosome exocytosis is slow and requires more stimulation. **α**-Granule exocytosis is considered to be intermediate. The kinetics and extent of platelet exocytosis vary depending on the concentration and potency of the agonist used, but whether the composition of released cargo follows any agonist-dependent patterns remains controversial ^71^. The distinct cellular localization of two major platelet v-SNAREs—VAMP-7 and VAMP-8, discussed in greater detail below—suggests a functional heterogeneity in granule exocytosis ^72, 73^. However, studies extensively characterizing cargo released using multiple agonists, employing both immunoassays and proteomics, suggest that there may not be any thematic patterns of cargo release ^74^. Thus, whether or not function-specific platelet exocytosis of α-granule subpopulations occurs under physiological conditions remains to be established.

Fusion of vesicle membrane with the plasma membrane is the general scheme of exocytosis in nucleated cells. Platelets follow this general rule but with some atypical features. Platelet granules, which are uniformly distributed throughout the platelet, move centrally upon platelet stimulation and spreading, although this may be artefactual. Second, in addition to fusion with the plasma membrane, most granule exocytosis follows fusion of platelet granules with the open canalicular system (OCS), which are plasma membrane invaginations that increase platelet surface area by at least two- to three-fold ^75, 76^. **α**-Granules fuse with the membrane individually as well as in the form of large multi-granular compartments that result from granule–granule fusion. This pattern of granule–granule fusion followed by granule–plasma membrane fusion occurs exclusively in **α**-granules at higher agonist concentrations ^77^.

### SNAREs

**Figure 2.**
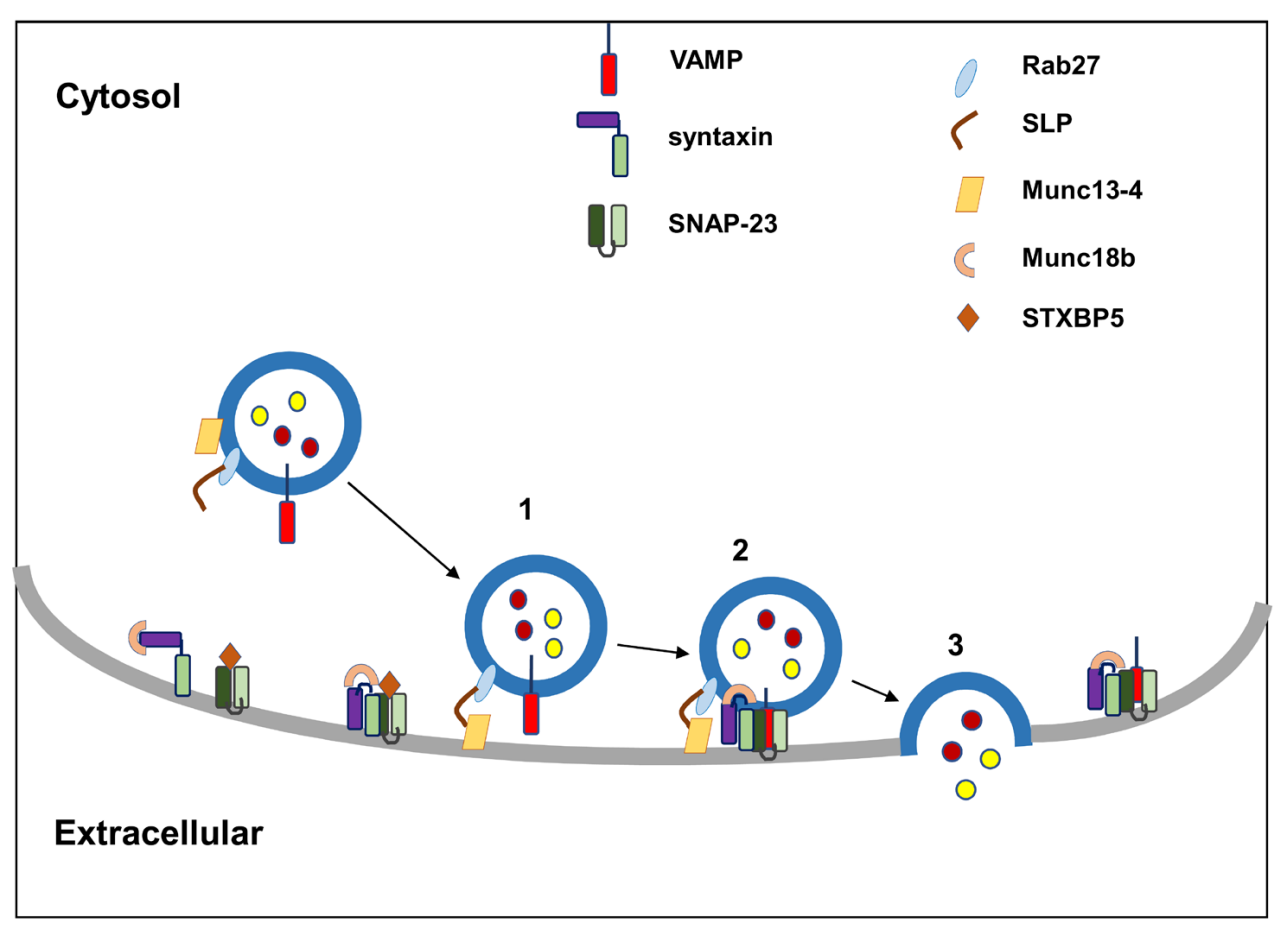
SNARE-mediated platelet granule exocytosis. The pathway of platelet granule exocytosis involves (1) granule docking, (2) priming, and (3) membrane fusion and cargo release. Rab27b and its effectors syntaptotagmin-like protein and Munc13-4 present on vesicle membrane are required for granule docking. Platelet activation promotes conformation change in syntaxins, sequestered by Munc18b in the resting state. This activation results in “priming” with subsequent formation of a four-helical bundle consisting of one R-SNARE provided by VAMP (red) and three Q-SNAREs provided by syntaxin and SNAP-23 (shades of green). In addition, syntaxin binding protein 5 (STXBP5) regulates t-SNARE function by binding syntaxin-SNAP-23 heterodimers. SNARE engagement ultimately leads to formation of the membrane fusion pore and cargo release. SNAP, soluble NSF attachment proteins; SNARE, soluble *N*-ethylmaleimide-sensitive factor attachment protein receptor; VAMP, vesicle-associated membrane protein.

Membrane fusion is facilitated by SNARE proteins, a family of highly conserved eukaryotic proteins essential for vesicle fusion ^78^. SNARE proteins are classified into two groups on the basis of their location: v-SNAREs, located on the vesicle/granule membrane, and t-SNAREs, located on the target membrane (for example, plasma membrane). Related v- and t-SNAREs interact through SNARE domains, which are **α**-helices of about 60 amino acids, assembled into amphipathic, heptad repeats. SNAREs can also be classified as R-SNAREs (typically v-SNAREs) or Q-SNAREs (typically t-SNAREs), depending on the presence of an arginine or glutamine residue, respectively, in the central position of the SNARE domain ^79^. Four SNARE domains—one each from the v-SNARE plus three t-SNAREs—form a coiled-coil structure that brings the two opposing membranes together (for example, granule and plasma membrane) against repulsive electrostatic forces of the two lipid membranes in an aqueous environment ( Figure 2) ^80^.

VAMPs constitute the largest group of v-SNAREs. Platelets contain VAMP-2, -3, -7, and -8; VAMP-8 is the most abundant and functionally important in human platelets, followed by VAMP-7 ^81^. Loss of VAMP-8 in mice causes defective **α**- and dense granule exocytosis, and platelet thrombus formation *in vivo*, without excessive bleeding ^82^. On the other hand, loss of VAMP-7 in mice leads to defective platelet spreading, and altered **α**- and dense granule exocytosis, without impacting platelet thrombus formation or bleeding ^73^. Moreover, VAMP-7 is located peripherally in the spreading platelet whereas VAMP-8 concentrates in the central granulomere ^72^. These observations suggest a distinct role for these v-SNAREs in platelet function. Interestingly, VAMP-8 has also been linked to early-onset myocardial infarction in genome-wide association studies, suggesting a syndrome of platelet hyper-responsiveness ^83^. VAMP-3, which is important in the endocytotic pathway of **α**-granule biogenesis, has minimal function in platelet exocytosis ^47^. The role of platelet-specific VAMPs has not been well established in granulogenesis.

Of the t-SNAREs, proteomic studies suggest that platelet contains syntaxin 2, 4, 6, 7, 8, 11, 12, 16, 17, and 18 and soluble NSF attachment proteins (SNAPs) 23, 25, and 29 ^84^. Of these, syntaxin 11 and SNAP 23 are the only t-SNAREs found to be essential for platelet granule exocytosis. As with v-SNAREs, most data come from mouse models lacking one or more specific t-SNAREs. Loss of syntaxin 11, which forms complexes with SNAP 23 and VAMP-8, is associated with abnormal exocytosis of all three of the major platelet granules ^85^. In humans, familial hemophagocytic lymphohistiocytosis type 4 is caused by lack of syntaxin 11 ^86^. Loss of syntaxin 8 has been associated with minor defects in dense granule exocytosis ^87^.

### SNARE regulators

To prevent indiscriminate release of granular content, fusion of vesicle and target membranes is tightly regulated by SNARE regulators. Some SNARE regulators are chaperones (for example, Munc18b), while others promote formation of membrane-fusion complexes and direct where the fusion occurs (for example, Munc13-4, Munc18 isoforms, Rabs, STXBP5/Tomosyn 1, and exocyst complex).

Munc18b is the most important syntaxin chaperone belonging to the Sec/Munc family of proteins present in the platelet, forming specific complexes with t-SNAREs. Munc18b deficiency leads to decreases in platelet levels of syntaxin 11, consistent with its role as a chaperone, resulting in impaired granule exocytosis ^88^. Homozygous deficiency, as seen in familial hemophagocytic lymphohistiocytosis type 5, leads to severe defects in all platelet granule exocytosis, whereas heterozygous deficiency leads to intermediate defects ^89^. VPS33B, discussed in α-granule biogenesis above, also belongs to the Sec/Munc family of proteins ^29^.

Syntaxin binding protein 5 (STXBP5), or tomosyn 1, binds to the cytoskeleton and to t-SNARE heterodimers (syntaxin 11 and SNAP-23) through the presence of a v-SNARE-like domain at its C-terminal. Its deficiency causes defective granule exocytosis, and mice lacking STXBP5 show excessive bleeding ^90^. Interestingly, STXBP5 negatively regulates VWF release from the endothelial cells, and polymorphisms in STXBP5 gene are associated with increased plasma VWF levels and cardiovascular disease ^91^.

Rab proteins that belong to the Ras superfamily of GTPases function as master regulators of the complex network of intracellular membrane trafficking pathways ^92^. Rabs perform this regulatory function by binding to effector proteins in the GTP-bound, or “on”, state ^93^. Some of these Rab effectors are SNARE regulators. Multiple Rabs, including Rab3b, 6c, and 8, are phosphorylated upon platelet activation, and their inhibition decreases platelet exocytosis ^94^. Among these, Rab4 is crucial for **α**-granule exocytosis whereas Rab27b is a key regulator of dense granule biogenesis and exocytosis ^95, 96^. Munc13-4 is a Rab27b effector protein, essential for dense granule function. Munc13-4 forms calcium-dependent bridges between the dense granule and plasma/OCS membrane, facilitating membrane fusion ^97, 98^. Rab27 ^-/-^ and Munc13-4 ^-/-^ platelets have defective dense granule exocytosis and a bleeding diathesis. These platelets also display defective exocytosis of **α**-granules and lysosomes, which can be overcome by the addition of ADP, a key dense granule component ^99^. This reversal by ADP, as also occurs in HPS platelets, demonstrates the critical role of autocrine signaling from released dense granule cargo for complete platelet activation ^15, 100^.

Synatotagmin-like proteins (SLPs), particularly SLP1 and SLP4, that bind calcium/lipids are also known to regulate dense granule exocytosis and may act as calcium sensors. SLP1—which forms a complex with Rap1, a Ras-like GTPase, and RAP1GEF2, its guanine nucleotide exchange factor—is a negative regulator of dense granule release ^101^. SLP4, a Rab27 effector, on the other hand, is a positive regulator of dense granule release ^102^.

Tethering complexes, particularly the exocyst complex, which is known to play a role in polarized secretion, may also be involved in the regulation of dense granule exocytosis ^103^. Exocyst complex is targeted to the plasma membrane by Ral, a Ras-like GTPase, which is expressed in platelets and activated upon platelet stimulation. Blocking of Ral-GTP binding to exocyst complex impairs dense granule exocytosis.

NSF and soluble NSF attachment proteins (SNAPs) are also important regulators of platelet exocytosis ^104^. These proteins disassemble SNARE complexes to allow recycling of v-SNAREs and t-SNAREs for the next round of membrane fusion ^105^. The inhibitory effect of nitric oxide on platelet exocytosis is at least partly due to its reversible inhibition of NSF ^106^.

Many SNAREs and their regulators, such as SNAP23 and Munc18, are known to be protein kinase C substrates, linking platelet activation and ensuing signaling cascades to the exocytosis machinery. Platelet signaling and protein phosphorylation and their role in regulated platelet exocytosis are beyond the scope of this review. The reader is referred to excellent reviews on this topic ^107– 109^.

## Conclusions

Regulated release of platelet granules is central to normal platelet function, which includes a variety of biological processes such as inflammation and immunity, in addition to hemostasis and thrombosis. Human platelet granule deficiency syndromes and their murine models, as well as the study of other cell types such as melanocytes and chromaffin cells ^92^, have been major sources of understanding of the protein machinery involved in platelet granulogenesis and exocytosis. Despite significant progress in identifying this machinery, many questions remain unanswered. What are the roles in granulopoiesis of the different vesicular trafficking proteins identified by genetic studies? What are the exact platelet-specific functions of SNARE regulators critical for platelet exocytosis, such as STXBP5, Munc13-4, and SLPs? Do platelet α-granules demonstrate a function-specific pattern of release, as may be inferred by evidence of different **α**-granule pools? How do secondary signaling mechanisms generated upon platelet activation control the distal exocytosis machinery? The answer to these questions will enable a clearer view of the life cycle of platelet granules, which is central to understanding platelet function in varied pathophysiologic processes.
